# The Medical Boards

**Published:** 1895-02

**Authors:** 


### *EDITORIAL.*

---

The office of THE JOURNAL is in room 330 Equitable Building

Address all communications, and make all remittances payable to THE ATLANTA MEDICAL AND SURGICAL JOURNAL, Atlanta, Ga.

Articles for publication should reach this office not later than the 15th instant.

The editors are not responsible for views expressed by contributors.

---

### THE MEDICAL BOARDS.

Since our last issue the governor has made the following appointments:

*Regular Board.*—Dr. F. M. Ridley, LaGrange, three years; Dr. J. B. Baird, Atlanta, one year; Dr. A. A. Smith, Hawkinsville, two years; Dr. E. R. Anthony, Griffin, two years; and Dr. W. A. O'Daniel, Milledgeville, one year.

*Homeopathic Board.*—Dr. M. A. Cleckley, Augusta, two years; Dr. R. A. Hicks, Rome, one year; Dr. John Z. Lawshe, Atlanta, one year; Dr. C. M. Paine, Atlanta, three years; and Dr. R. E. Hinman, Atlanta, two years.

*Eclectic Board.*—Dr. J. Frank Harris, Thomas county, three years; Dr. John F. Harris, Dalton, two years; Dr. M. K. Phillips, Bremen, two years; Dr. M. T. Salter, Atlanta, one year; and Dr. W. V. Robertson, Morgan county, one year.

January 22d, all the boards met in Atlanta for organization. The regular board elected Dr. A. A. Smith President, and Dr. F. M. Ridley Secretary and Treasurer. The homeopathic board elected Dr. M. A. Cleckley President, and Dr. R. E. Hinman Secretary and Treasurer. The eclectic board elected Dr. J. Frank Harris President, and Dr. M. T. Salter Secretary and Treasurer. The second meetings of the boards will be held in the spring, the dates yet to be appointed.

We believe these to be excellent appointments, and it only remains for the boards to carry out the spirit and purpose of the law to protect our people and the State medical profession from the hordes of frauds and incompetents who have been far too numerous in Georgia heretofore. The meetings of the boards showed that they were in earnest about their work, and we do not doubt they will do their whole duty.

